# Diagnostic Performance of the Rapid Antigen Test as a Screening Tool for SARS-CoV-2 Infection in the Emergency Department

**DOI:** 10.3390/jpm12071172

**Published:** 2022-07-19

**Authors:** Heekyung Lee, Hyunggoo Kang, Yongil Cho, Jaehoon Oh, Tae-Ho Lim, Byuk-Sung Ko, Juncheol Lee

**Affiliations:** Department of Emergency Medicine, College of Medicine, Hanyang University, Seoul 04763, Korea; massdt@hanyang.ac.kr (H.L.); emer0905@gmail.com (H.K.); ojjai@hanyang.ac.kr (J.O.); erthim@hanyang.ac.kr (T.-H.L.); postwinston@gmail.com (B.-S.K.); doldoly@hanyang.ac.kr (J.L.)

**Keywords:** SARS-CoV-2 infection, COVID-19, emergency department, rapid antigen test, cyclic threshold values

## Abstract

The rapid antigen test (RAT) has been adopted as a screening tool for SARS-CoV-2 infection in many emergency departments (EDs). We aimed to investigate the diagnostic value of the accuracy of the SARS-CoV-2 RAT as a screening tool in the ED. This retrospective observational study included patients who underwent both RAT and RT–PCR and visited the ED from 1 December 2021 to 15 March 2022. RAT and RT–PCR were performed by appropriately trained physicians. We performed detailed analyses using the E gene cyclic threshold (Ct) values of RT–PCR. Out of a total of 1875 patients, 348 (18.6%) had positive and 1527 (81.4%) had negative RT–PCR results. The overall sensitivity, specificity, positive predictive value, and negative predictive value of the RAT were 67.8%, 99.9%, 99.6%, and 93.2%, respectively. The E gene Ct value was significantly lower in the RAT-positive patients than in the RAT-negative patients (18.5 vs. 25.3, *p* < 0.001). When the E gene Ct cutoff was 30.0, 25.0, 20.0, and 15.0, the sensitivity of the RAT was 71.9%, 80.3%, 93.0%, and 97.8%, respectively. The sensitivity of the RAT could be considered high in patients with a high viral load, and the RAT could be used as a screening tool in the ED.

## 1. Introduction

The outbreak of severe acute respiratory syndrome coronavirus 2 (SARS-CoV-2) was initiated in China in late December 2019, and coronavirus disease 2019 (COVID-19) has progressed as a global pandemic [1,2]. As of June 2022, there have been more than 500 million confirmed cases and more than 6 million cumulative deaths worldwide due to COVID-19 [3]. The variants and mutations of SARS-CoV-2 continue to be discovered and have led to the continuation of the pandemic [4].

The early identification and isolation of COVID-19 patients to prevent the spread of SARS-CoV-2 infection is crucial, especially in the emergency department (ED) [5]. Patients with various pathological conditions, including critically ill patients who visit the ED, and a large number of elderly patients with underlying disease stay in the ED [6]. The result of failure to accurately identify patients with SARS-CoV-2 infection can be fatal, since those patients have a higher mortality rate from SARS-CoV-2 infection [7]. Additionally, if a patient with COVID-19 is admitted to a non-isolated ward in the ED without recognition of infection, nosocomial spread may occur [8].

Real-time reverse transcription polymerase chain reaction (RT–PCR) is currently used as a diagnostic gold standard for the detection of SARS-CoV-2 infection [9,10]. Nucleic acid amplification tests (NAATs, such as RT–PCR) have high sensitivity and specificity for detecting SARS-CoV-2 infection and can be performed on upper respiratory specimens [11]. However, RT–PCR has limitations in that the test requires specialized equipment with expertise, the turn-around time is approximately 1 to 3 days, and the cost is higher [10]. In the ED situation, the time required to confirm the test result can cause a delay in assessing patients, which causes unnecessary damage [12,13,14]. Rapid antigen tests (RATs) are immunoassays that detect a specific viral antigen and can be used as a screening test. The Ag test can provide rapid results within 15 to 30 min, and the cost per test is lower than that of other tests [15]. Point-of care screening tests in the ED are especially important when there is a high number of patients with COVID-19 in the community [16]. However, a confirmation test is needed because of the lower sensitivity of RAT compared to that of NAATs [17,18].

The RAT has been adopted as a screening tool for SARS-CoV-2 infection in many EDs [19,20,21,22]. However, the reported sensitivity of the RAT varies depending on the study design and the population. Additionally, its value as a screening tool for ED settings is still unclear and debatable. We aimed to investigate the diagnostic value and the accuracy of the SARS-CoV-2 RAT as a screening tool in the ED.

## 2. Materials and Methods

### 2.1. Study Design and Population

This retrospective observational study was conducted in the ED of Hanyang University Hospital in Seoul, Korea. This study was approved by the Institutional Review Board (IRB) of Hanyang University Hospital (IRB No. HYUH 2022-03-037). The requirement for informed consent was waived because the data were entirely retrospectively collected.

We included patients who visited the ED from 1 December 2021 to 15 March 2022. From December 2021, we introduced a protocol to perform RAT on almost all patients who visited the ED. The identification of viral RNA using RT–PCR is the gold standard for identifying SARS-CoV-2 infection. Patients who underwent RAT and concurrent COVID-19 PCR were included in the study. We performed RT–PCR along with RAT for ED patients with symptoms or signs related to COVID-19 or who needed hospitalization.

### 2.2. Setting and Sample

RAT and RT–PCR swabs for COVID-19 were performed by appropriately trained physicians. RAT was performed using samples collected from nasopharyngeal swabs. RAT was performed with the STANDARD™ Q COVID-19 Ag Test (SD BIOSENSOR, Gyeonggi-do, Korea). RAT is a rapid chromatographic immunoassay for the qualitative detection of SARS-CoV-2 nucleocapsid antigen. The COVID-19 antigen test cassette is coated with two lines: a control line and a test line. The control line was coated with mouse monoclonal anti-chicken IgY antibody, and the test line was coated with mouse monoclonal anti-COVID-19 antibody. If the SARS-CoV-2 antigen is present in the sample, the purple line is displayed on the test line. The control line is always displayed when the sample migration is successful. Visual interpretation of the results was performed between 15 and 30 min.

RT–PCR was performed using samples collected from both nasopharyngeal and oropharyngeal swabs. Two RT–PCR assays were conducted as a reference standard test to confirm the presence of COVID-19. PowerCheck™ 2019-nCoV RT–PCR (Kogene Biotech, Seoul, Korea) was performed in hospital during weekdays. The AllplexTM 2019-nCoV Assay (Seegene, Seoul, Korea) was performed as an outsourced test at night and on weekends. The cyclic threshold (Ct) cutoff indicating COVID-19 positivity was ≤38 for the PowerCheck™ 2019-nCoV RT–PCR and ≤40 for the AllplexTM 2019-nCoV Assay. The Ct value of COVID-19 has been reported for the E, RdRP/S, and N genes; E and ORF1ab gene Ct values in Omicron patients. Therefore, the E gene Ct values in patients with COVID-19 have always been reported, regardless of whether they were Omicron patients. For this reason, we performed various detailed analyses using the E gene Ct values.

### 2.3. Statistical Analysis

Categorical variables are presented as numbers and percentages. Fisher’s exact test was performed when comparing the categorical variables of two groups. The normality test of continuous variables was performed using the Shapiro–Wilk test. Continuous variables with normality are presented as the mean and standard deviation, and variables with nonnormal distribution are presented as the median and 25th to 75th percentiles. Student’s *t*-test or Wilcoxon rank sum test was performed depending on the results of the normality test.

Sensitivity, specificity, positive predictive value (PPV), and negative predictive value (NPV) were calculated using 2 × 2 tables for the RAT and RT–PCR test results and are presented as % and 95% confidence intervals; this was calculated using the EpiR library of the R program. Among the confirmed COVID-19 patients, we identified the sensitivity of RAT according to various E gene Ct cutoff values of RT–PCR. On admission, the sensitivity of RAT was investigated when each Ct value interval of the E gene was defined for COVID-19-positive patients. Statistical significance was determined when the *p* value was less than 0.05 on a two-sided test. All statistical analyses were conducted using R (version 4.2.0, R Foundation for Statistical Computing, Vienna, Austria).

## 3. Results

We included 1875 patients who had RAT and RT–PCR concurrently and who visited the emergency department. The baseline characteristics of the study population are summarized in Table 1. Of these, 348 patients (18.6%) had positive COVID-19 RT–PCR results, and their median age was 49.5 (25–75th percentile: 26–74). A total of 1527 patients had negative COVID-19 RT–PCR results, and their median age was 55 (25–75th percentile: 28–72). The proportions of females in the COVID-19-positive and -negative patient groups were 49.7% and 53.6%, respectively, and there was no significant difference between the two groups. The median body temperature of the COVID-19-positive patients was 37.7 °C, which was significantly higher than the 37.0 °C of the COVID-19-negative patients. The RAT was positive in 237 (12.6%) patients and negative in 1638 (87.4%) patients.

**Figure 1:**
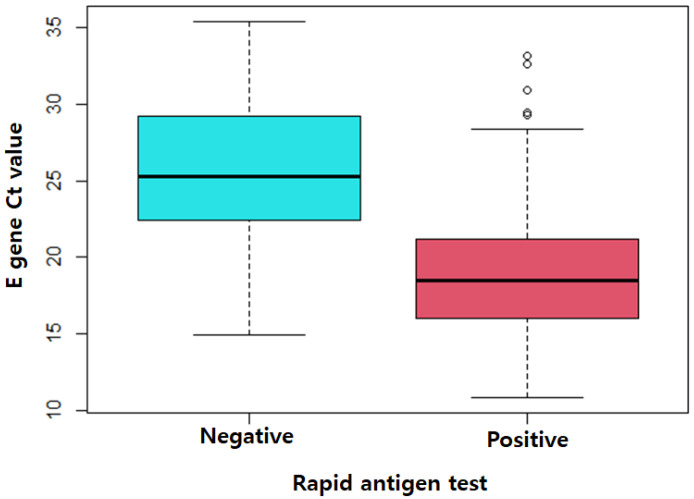
E gene Ct value of RT–PCR according to the rapid antigen test results in COVID-19 patients (*n* = 348).

The overall sensitivity of RAT was 67.8% (95% confidence interval (CI), 62.6–72.7); the specificity was 99.9% (95% CI, 99.6–100.0) (Table 2). The number of false-negative RAT results was 112, and the NPV was 93.2% (95% CI, 91.8–94.3). The number of false-positive RAT results was 1, and the PPV was 99.6% (95% CI, 97.7–100.0). The median E gene Ct value of the COVID-19 patients was significantly lower in the RAT-positive patients (median, 18.5; 25–75th percentile, 16.0–21.2) than in the RAT-negative patients (median, 25.3; 25–75th percentile, 22.4–29.2) (*p* value < 0.001), suggesting a high viral load (Figure 1).

**Figure 2:**
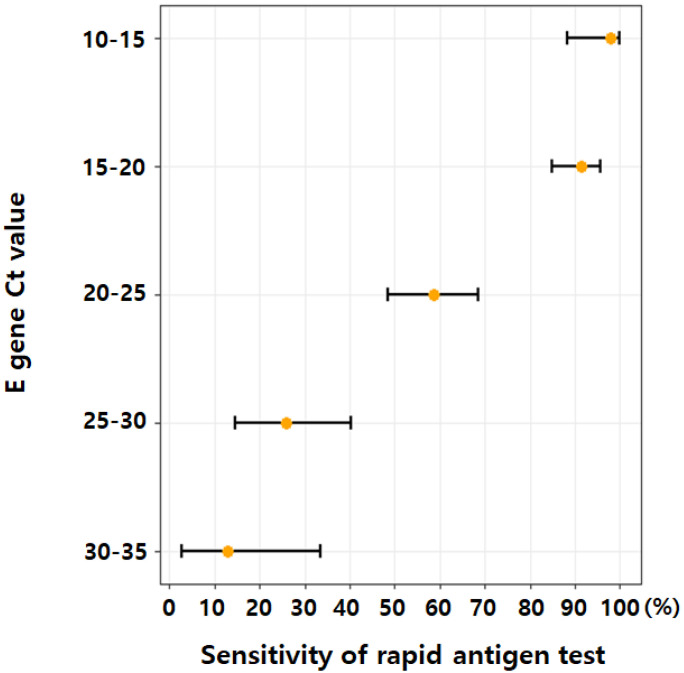
Sensitivity of RAT according to the E gene Ct value interval defining COVID-19 positive confirmation.

We examined the sensitivity of RAT according to the E gene Ct cutoff value of RT–PCR in COVID-19 patients (Table 3). When the median E gene Ct value (20.1) was used as the adapted cutoff for COVID-19 positivity, the sensitivity of RAT was 96.5% (95% CI, 88.3–96.4); when the first quartile (17.1) was used as the cutoff, it was 96.5% (95% CI, 90.1–99.3). When the E gene Ct cutoff was 30.0, 25.0, 20.0, and 15.0, the sensitivity of RAT was 71.9 (66.7–76.7), 80.3 (75.1–84.8), 93.0 (88.1–96.3), and 97.8 (88.2–99.9), respectively. We examined the sensitivity of RAT according to the E gene Ct value interval that defines COVID-19 positive confirmation (Figure 2). The sensitivity of RAT for Ct values 10–15, 15–20, 20–25, 25–30, and 30–35 was 97.8% (95% CI, 88.2–99.9), 91.3% (95% CI, 85.0–95.6), 58.8% (95% CI, 48.6–68.5), 26.0% (95% CI, 14.6–40.3), and 13.0% (95% CI, 2.8–33.6), respectively.

## 4. Discussion

In the current study, we aimed to investigate the diagnostic performance of the SAR-CoV-2 Ag test as a screening tool in the ED. The RAT showed high specificity, PPV and NPV, and low overall sensitivity (67.8%). The sensitivity was strongly correlated with the Ct value and ranged from 14.3% (Ct > 30.0) to 97.8% (Ct ≤ 15.0).

The NAATs and the antigen test are used as viral tests to detect SAR-CoV-2 infection and as screening tests to decrease the spread of SAR-CoV-2 by identifying and isolating infected individuals [10]. NAATs are highly sensitive and specific tests and can detect a recent infection with a prolonged viral RNA detection. NAATs are usually performed in a laboratory, and the turn-around time ranges from 1 to 3 days [10]. Antigen tests are immunoassays that detect the presence of a specific viral antigen. The characteristic of antigen testing, which is cheaper, faster, and easier to perform, makes it a useful tool to identify individuals with SAR-CoV-2 infection. However, the lower sensitivity than that of NAATs requires confirmation tests, such as RT–PCR, in specific circumstances [17,18].

According to the current guidance from the World Health Organization, the RAT can offer a faster and less expensive method to diagnose active SARS-CoV-2 infection than NAATs [18]. The diagnostic performance of the RAT is higher in infected individuals with a high viral load and in the early phase of infection [18]. The interim guidance recommends the RAT when it fulfills a minimum sensitivity of 80%, and careful selection of cohorts could mitigate the low sensitivity of the RAT [18]. Since the RAT was commercialized, research on the utility of the RAT as a screening tool has been widely conducted and reported [23]. A recent meta-analysis reported that the overall sensitivity of the RAT based on 94 studies was 70%, and RAT could be a viable option in the early phase of infection when laboratory facilities are lacking [9]. Turcato et al. reported that the RAT could improve the identification of patients with COVID-19 infection in the ED, especially symptomatic patients [21]. However, another study concluded that the RAT is not sufficient to use alone as frontline testing for the diagnosis of COVID-19 due to its poor sensitivity of 30.2% [17]. One prospective cross-sectional study for a primary/secondary care testing facility revealed that the sensitivity of the RAT was lower than that of the manufacturer’s data, which could lead to falsely classifying individuals with a negative result [24].

The Ct value could be used as a marker for predicting the contagion and viral load of an individual, and a significant relationship between a low Ct value and a high risk of secondary transmission was reported [25]. Additionally, the sensitivity of RAT for SARS-CoV-2 was 96% when the Ct value was less than 25 in the subgroup analysis according to a recent meta-analysis [26]. In the present study, the sensitivity was 80.3% and 97.8% when the Ct value was lower than 25.0 and 15.0, respectively. These results suggest that the RAT would be more reliable in high viral loads and highly contagious individuals who could spread infection.

The length of stay (LOS) and crowding are significantly associated with the outcomes of patients in the ED [27,28]. During the COVID-19 pandemic, the ED LOS was significantly longer than the pre-pandemic LOS despite the decreased number of patients visiting the ED in many countries [12,13,14]. Furthermore, an increased length of stay could lead to crowding in the resuscitation area in the pandemic period [13]. The limited number of isolation areas or resuscitation areas causes delays for the patients admitted to the ED, especially when there are a high number of patients with COVID-19 in the community. The reference diagnosis, NAATs, could worsen the ED LOS and prolong the immediate decision making for patients by ED physicians. The strengths of the RAT, which are its simplicity, high specificity, rapid performance, and immediate confirmation of results, could overcome the low sensitivity and be suitable for ED situations. The early recognition of the infected individual on the frontline of the ED could reduce the LOS for ED admission and overcrowding. Additionally, patients with SAR-CoV-2 infection could be immediately provided with appropriate management, considering the high specificity and low false positive rate.

The current study has several limitations. First, the present study was conducted in a single center, and the study had a retrospective design. These factors could affect the results of the study, and if this study had included individuals from countries with different healthcare systems and races, the results may have been different. Second, we performed the RAT with one product (STANDARD™ Q COVID-19 Ag Test, SD BIOSENSOR, Korea). The sensitivity and specificity could differ when using other RAT commercial products. Third, although we tried to analyze variables, there could be hidden confounders that could affect the results. Fourth, RT–PCR was conducted within 48 h in all included individuals; however, the time gap between RT–PCR and RAT was not analyzed. Fifth, during the study period of the current study, the COVID-19 community level was extremely high. This could affect the results of the study.

## 5. Conclusions

The sensitivity of RAT could be considered high in patients with a high viral load. The RAT could be used as a screening tool in the ED, especially when the number of COVID-19 patients is high in the community. Future prospective studies are needed to enhance the findings of the current study.

## Figures and Tables

**Table 1:** Baseline characteristics of the study population.

	COVID-19 RT–PCR		
	Positive (*n* = 348)	Negative (*n* = 1527)	*p* Value *
Age, median [25–75th percentile]	49.5 (26–74)	55 (28–72)	0.410
Age distribution, *n* (%)			0.053
<20	56 (16.1)	183 (12.0)	
≥20 to <65	162 (46.6)	798 (52.3)	
≥65 to <75	46 (13.2)	211 (13.8)	
≥75 to <85	47 (13.5)	222 (14.5)	
≥85	37 (10.6)	113 (7.4)	
Sex, *n* (%)			0.342
Male	175 (50.3)	724 (47.4)	
Female	173 (49.7)	803 (52.6)	
Temperature at admission (°C)	37.7 [36.9–38.4]	37.0 [36.5–37.7]	<0.001
Rapid antigen test, *n* (%)			<0.001
Positive	236 (67.8)	1 (0.1)	
Negative	112 (32.2)	1526 (99.9)	

* The Wilcoxon rank sum test was performed for continuous variables. Fisher’s exact test was performed for categorical variables.

**Table 2:** Diagnostic performance of the rapid antigen test in the study population.

	%	95% CI
Sensitivity	67.8	62.6–72.7
Specificity	99.9	99.6–100.0
PPV	99.6	97.7–100.0
NPV	93.2	91.8–94.3

CI = confidence interval; PPV = positive predictive value; NPV = negative predictive value.

**Table 3:** Sensitivity of the rapid antigen test classified according to the adapted E gene Ct cutoff value of the RT–PCR in COVID-19 patients.

E Gene Ct Value	Number of COVID-19 Positive Patients	Sensitivity (%) [95% CI]
Ct value category 1		
Ct ≤ 24.3 ^a^	259	82.2 [77.0–86.7]
Ct ≤ 20.1 ^b^	174	93.1 [88.3–96.4]
Ct ≤ 17.1 ^c^	86	96.5 [90.1–99.3]
Ct value category 2 ^d^		
Ct ≤ 30.0	324	71.9 [66.7–76.7]
Ct ≤ 25.0	274	80.3 [75.1–84.8]
Ct ≤ 20.0	172	93.0 [88.1–96.3]
Ct ≤ 15.0	45	97.8 [88.2–99.9]

CI = confidence interval, ^a^ Third quartile, ^b^ Median, ^c^ First quartile, ^d^ Intervals in increments of 5.

## Data Availability

The datasets used and/or analyzed during the current study are available from the corresponding author on reasonable request.
